# The Effect of Fine and Coarse Particulate Air Pollution on Mortality: A National Analysis

**DOI:** 10.1289/ehp.0800108

**Published:** 2009-02-13

**Authors:** Antonella Zanobetti, Joel Schwartz

**Affiliations:** Department of Environmental Health, Harvard School of Public Health, Boston, Massachusetts, USA

**Keywords:** cardiovascular diseases, fine particulate matter, mortality, PM coarse, respiratory disease, season, time series

## Abstract

**Background:**

Although many studies have examined the effects of air pollution on mortality, data limitations have resulted in fewer studies of both particulate matter with an aerodynamic diameter of ≤ 2.5 μm (PM_2.5_; fine particles) and of coarse particles (particles with an aerodynamic diameter > 2.5 and < 10 μm; PM coarse). We conducted a national, multicity time-series study of the acute effect of PM_2.5_ and PM coarse on the increased risk of death for all causes, cardiovascular disease (CVD), myocardial infarction (MI), stroke, and respiratory mortality for the years 1999–2005.

**Method:**

We applied a city- and season-specific Poisson regression in 112 U.S. cities to examine the association of mean (day of death and previous day) PM_2.5_ and PM coarse with daily deaths. We combined the city-specific estimates using a random effects approach, in total, by season and by region.

**Results:**

We found a 0.98% increase [95% confidence interval (CI), 0.75–1.22] in total mortality, a 0.85% increase (95% CI, 0.46–1.24) in CVD, a 1.18% increase (95% CI, 0.48–1.89) in MI, a 1.78% increase (95% CI, 0.96–2.62) in stroke, and a 1.68% increase (95% CI, 1.04–2.33) in respiratory deaths for a 10-μg/m^3^ increase in 2-day averaged PM_2.5_. The effects were higher in spring. For PM coarse, we found significant but smaller increases for all causes analyzed.

**Conclusions:**

We conclude that our analysis showed an increased risk of mortality for all and specific causes associated with PM_2.5_, and the risks are higher than what was previously observed for PM_10_. In addition, coarse particles are also associated with more deaths.

Many multicity studies have shown that ambient particulate air pollution, generally measured as particulate matter with aerodynamic diameter ≤ 10 μm (PM_10_), is associated with increased risk of death for broadly defined cardiovascular or respiratory causes, using time series analysis (Dominici et al. 2000; Katsouyanni et al. 1997; Schwartz 1994, 2000) or case–crossover analysis (Schwartz 2004; Zeka et al. 2005).

It is generally thought that fine particles (those with an aerodynamic diameter of ≤ 2.5 μm; PM_2.5_) are more harmful to health than larger particles (PM_10_) (Cifuentes et al. 2000; Schwartz et al. 1996), although some studies have shown stronger effects with coarse particles (particles 2.5–10 μm in aerodynamic diameter) (Ostro et al. 2000).

The literature on the association between fine particles (PM_2.5_) and mortality is relatively sparse, because of two main issues: The U.S. Environmental Protection Agency (EPA) began PM_2.5_ monitoring in 1999, and mortality data from the National Center for Health Statistics (NCHS) were not available nationwide after the year 2000. Nevertheless, several multicity studies have been performed. Using early PM_2.5_ monitoring data from the Harvard Six City Study, Schwartz et al. (1996) reported a strong association between 2-day average PM_2.5_ and daily deaths, but little association with coarse particles. A study of eight Canadian cities similarly found associations with fine but not coarse particles (Burnett et al. 2000). Dominici et al. (2007) examined PM_2.5_ mortality associations using national data in the United States, but only for the years 1999–2000.

Franklin and co-authors in two papers (Franklin et al. 2007, 2008) addressed this issue a different way. They used mortality data up to year 2000 from the NCHS, whereas they obtained mortality data directly from state health departments for the years 2001–2005 to examine the mortality effects of PM_2.5_ for an extended period of time. They were limited by their ability to obtain mortality state data, however.

Ostro et al. (2006) examined the associations between PM_2.5_ and daily mortality for the years 1999–2002 in nine California counties. They also found significant associations with PM_2.5_.

With a common effort of the U.S. EPA–funded PM research centers and the help of the U.S. EPA, we were able to obtain mortality data from 2001 through 2005 from each state in the country (except Hawaii and Idaho) through NCHS. As a result, national data for an extended period are available for the first time.

Based on the associations found in the previous studies and the new mortality data available, we hypothesized that PM_2.5_ is associated with increased risk of deaths in a national study. Because coarse particles are not currently regulated by the U.S. EPA, we also test the hypothesis that PM coarse (PM with aerodynamic diameter > 2.5 and < 10 μm) are associated with mortality. We therefore conducted a multicity time series study of the acute effect of PM_2.5_ and PM coarse on the increased risk of death for all causes, all cardiovascular disease (CVD), myocardial infarction (MI), stroke, and respiratory mortality.

## Materials and Methods

### Health data

The NCHS provided researchers with national data sets of mortality records, including date of death. Certain locations were excluded from the data, either because the population of the county was low, or because the state was not willing to cooperate with the project. The analysis was conducted at the county level, because this was the smallest resolution available for all mortality data; the name of the major city within each county was used as an identifier rather than the county name. The mortality data used provided non-confidential information on decedents including state of death, county of death, age, sex, date of death and primary cause of death.

We excluded those individuals who died in a state different from their state of residence. Only those individuals who died of nonaccidental causes were examined [i.e., *International Statistical Classification of Diseases, 10th Revision* (ICD-10; World Health Organization 2007) codes S00 through U99 were excluded].

Specific causes were derived from the ICD-10 code for the underlying cause of death: respiratory disease (ICD-10: J00 through J99), CVD (ICD-10: I01 through I59), MI (ICD-10: I21 through I22), stroke (ICD-10: I60 through I69), and all-cause mortality (TOT; ICD-10: A00 through R99).

For all nonaccidental deaths and for each specific cause, we created daily counts of deaths in each of the examined counties. This work was done under an exemption from Human Subjects Committee of the Harvard School of Public Health.

### Environmental data

We obtained data on PM_2.5_ and PM_10_ from the U.S. EPA Air Quality System Technology Transfer Network (U.S. EPA 2008), which provides daily PM_2.5_ concentrations from the U.S. EPA National and State Local Ambient Monitoring stations.

In most cities, the analysis was conducted on a county level, because the city lies within a single county. However, we used multiple counties for Minneapolis–St. Paul, Minnnesota (Ramsey and Hennepin), Boston, Massachusetts (Middlesex Norfolk, Suffolk), Birmingham, Alabama (Blount, Jefferson, Shelby, St. Clair, Walker), Atlanta, Georgia (Cobb, De Kalb, Fulton, Gwinnett), Miami, Florida (Dade, Miami–Dade), Baltimore, Maryland (Baltimore City, Baltimore County), St. Louis, Missouri (Jefferson, Madison, St. Louis, St. Louis City, St. Clair), New York City, New York (Kings, New York City, Queens, Richmond), Steubenville (Jefferson, OH; Brooke, Hancock, WV) and Youngstown, Ohio (Mahoning, Columbiana), San Antonio, Texas (Bexar, Comal), Norfolk, Virginia (Newport News city, Norfolk city, Virginia Beach city), Kansas City, Missouri and Kansas (Jackson, Clay, MO; Johnson, KS), and Washington, DC (Arlington, VA; Washington, DC), where the city’s population extends beyond the boundaries of one county.

When more than one monitor was available in one county, the 24-hr integrated mass concentrations were averaged over the county using a method previously described (Schwartz 2000; Zanobetti et al. 2000). Briefly, we first excluded any monitor that was not well correlated with the others (*r* < 0.8 for two or more monitor pairs within a county), because it likely measured a local pollution source and would not represent the general population exposure over the entire community. We then computed the annual mean for each monitor and year and subtracted that mean from the daily values of that monitor. We then standardized these daily deviances by dividing by the standard deviation for that monitor. The daily standardized deviations for each monitor on each day were averaged, producing a daily averaged standardized deviation. We finally multiplied this by the standard deviation of all of the monitor readings for the entire year and added back in the annual average of all of the monitors. This process automatically compensates for missing data in some monitors on individual days by preventing that missingness from contributing to false variations in the daily value. This process has been reported previously (Schwartz 2000) and used extensively in previous publications (O’Neill et al. 2003; Wellenius et al. 2006; Zanobetti and Schwartz 2005; Zanobetti et al. 2000; Zeka et al. 2005).

**Figure 1:**
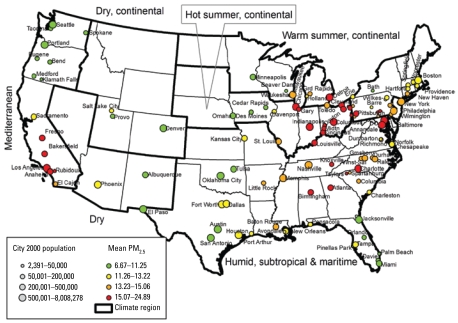
Map of the 112 U.S. cities included in the study. Symbol size represents the population; color represents PM_2.5_ concentrations.

To be included in our study, we required that at least 265 days of data in at least 1 year be available. We found 112 cities with at least 265 days of monitoring of PM_2.5_ per year and at least 300 days of mortality data per year from NCHS. They represented a geographic distribution across the country [Figure 1; Table 1 in Supplemental Material (available online at http://www.ehponline.org/members/2009/0800108/suppl.pdf)]. PM coarse was estimated by differencing the countywide averages of PM_10_ and PM_2.5_. PM coarse was available for fewer locations, because less monitoring of PM_10_ is currently being done. We had 47 locations that met our criteria for PM coarse.

We obtained local meteorologic data from the U.S. Surface Airways and Airways Solar Radiation hourly data (National Environmental Satellite, Data and Information 2003).

Because climate may effect exposure to particles (e.g., because ventilation varies by climate) and may also modify particle characteristics (e.g., because climate affects secondary particle formation), we divided the United States into regions based on the Köppen climate classification (Kottek 2006; Kottek et al. 2006), which is one of the most widely used climate classification systems. The Köppen climate classification scheme divides the climates into five main groups and several types and subtypes. We used the following classification: region 1: humid subtropical climates and maritime temperate climates, which includes Florida, Louisiana, Texas, Georgia, Alabama, Mississippi, Arkansas, Oklahoma, Kansas, Missouri, Tennessee, South Carolina, North Carolina, Virginia, West Virginia, Kentucky; region 2: warm summer, continental climates, including North Dakota, Minnesota, Wisconsin, Michigan, Pennsylvania, New York, Connecticut, Rhode Island, Massachusetts, Vermont, New Hamphsire, Maine; region 3: hot summer, continental climates with South Dakota, Nebraska, Iowa, Illinois, Indiana, Ohio; region 4: dry climates, New Mexico, Arizona, Nevada; region 5: dry climates together with continental climate with Montana, Idaho, Wyoming, Utah, Colorado; region 6: Mediterranean climates, which includes California, Oregon, Washington.

### Analytical strategy

We investigated the association between PM_2.5_ and PM coarse concentrations averaged over the day of death and day before death and mortality with a time series analysis. The analysis was stratified by season because the composition of particles varies seasonally (partly due to different source contributions at different times of the year) and because the penetration of outdoor particles indoors varies seasonally.

We first applied season- and city-specific Poisson regression models, controlling for long-term trend and seasonality with a natural cubic regression spline with 1.5 degrees of freedom for each season for each year; day of the week using indicator variables; and for weather using a natural cubic spline with three degrees of freedom for the same-day temperature and for the previous-day temperature.

We first applied single-pollutant models and then fit a model including both PM_2.5_ and PM coarse. To test the hypothesis that coarse particles may affect mortality with a longer lag, making the choice of lags 0 and 1 less appropriate, we also fit a distributed lag model for 4 days, from the same day and up to 3 days earlier for coarse particles.

The city-specific results were then combined with a random effects meta-analysis (Berkey et al. 1998). To be conservative, we report the results incorporating a random effect, whether or not there was a significant heterogeneity.

The pooled analysis was done for each outcome separately, with a total of 448 city- and season-specific coefficients for PM_2.5_ and 188 for PM coarse. In the two-pollutant model, the meta-analysis was done for each pollutant separately.

We used the I^2^ statistic to assess the proportion of total variation in effect estimates that was attributed to between-city heterogeneity (Higgins and Thompson 2002). The I^2^ statistic is a generalization of the C^2^ or Q test for heterogeneity and expresses the proportion of variance explained. We used the following formula: I^2^ = [Q/(k−1)] −1/[Q/(k−1)], where Q is the Q-test for heterogeneity and k is the number of community. If Q/(k−1) is < 1, then the I^2^ is null and indicates that no variability is attributable to heterogeneity (zero in the tables).

We analyzed the data using R version 2.7.2 (R Development Core Team 2008). The effect estimates were expressed as a percent increase in mortality for a 10-μg/m^3^ increase in PM_2.5_ mass or PM coarse concentration.

## Results

In the 112 cities during the study period 1999–2005, there were 5,609,349 total deaths, 1,787,078 for CVD, 397,894 for MI, 330,613 for stroke, and 547,660 for respiratory disease. The biggest cities are Los Angeles, California; New York City, New York; and Chicago, Illinois.

The cities with higher levels of PM_2.5_ were in California, with maximum concentrations of PM_2.5_ > 100 μg/m^3^, whereas the cities with the lower maximum levels were in Oklahoma. The median PM_2.5_ ranged from 5.6 μg/m^3^ in Albuquerque, New Mexico, followed by Eugene and Bend, Oregon, with 6 μg/m^3^, whereas the highest median concentrations were 21.5 μg/m^3^ in Rubidoux, California, and 17.4 μg/m^3^ in Los Angeles.

Table 1 in Supplemental Material (available online at http://www.ehponline.org/members/2009/0800108/suppl.pdf) presents the following for each of the 112 cities: the years of study, the daily mean concentration levels for PM_2.5_ and PM coarse, and the daily mean number of death by cause.

Figure 1 shows the location of the 112 U.S. cities included in the study; the symbol size represents the population in each city, and the color represents the PM_2.5_ concentrations. High levels of PM_2.5_ (red) are in California and in the industrial Midwest.

Table 1 shows the percent increase in mortality for a 10-μg/m^3^ increase in PM_2.5_ for the mean of lags 0 and 1 (henceforth mean01), for the mean01 by season.

We found significant associations with all the analyzed causes of death and PM_2.5_, with the highest effect for stroke with a 1.78% increase [95% confidence interval (CI), 0.96–2.62], and respiratory mortality with a 1.68% increase (95% CI, 1.04–2.33) for a 10-μg/m^3^ increase in the mean01 of PM_2.5_. When looking at the results by season, the highest effects are in spring, with > 2% increases.

Table 2 shows the percent increase in mortality for a 10-μg/m^3^ increase in PM coarse across the 47 cities for the sum of the 4 days distributed lag model and by season. We found significant associations with total mortality, stroke, CVD, and respiratory mortality, for which we had the largest effect: a 1.2% increase (95% CI, 0.4–1.9) for a 10-μg/m^3^ increase in PM coarse. The effect sizes per unit of mass for PM coarse are about half those for PM_2.5_.

**Figure 2:**
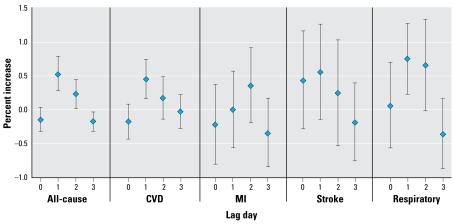
Percent increase in cause-specific mortality for the 4 days distributed lag model, combined in 47 cities for PM coarse. Error bars represent 95% CIs of the estimates.

When we examined the distributed lag for PM coarse (Figure 2), we found little evidence that the effects were at longer lags. Figure 2 reports the percent increase in cause-specific mortality at each lag from the distributed lag model, combined across the 47 cities. There were indications of some effect at lag 2, but the sum of the distributed lag was not higher than the results for mean01.

When looking at the heterogeneity by season, we found no significant heterogeneity in general in summer (Table 1), whereas significant heterogeneity was seen in spring and autumn. For all-cause mortality and PM_2.5_, 32% in spring and 15% in autumn of the total variability in city-specific coefficients was attributable to between-community differences (as opposed to stochastic variation). For PM coarse, we only found significant city-specific heterogeneity for all-cause and respiratory mortality. Again, the highest percentage of explained total variability was in spring.

Table 3 shows the results from the multipollutant model, which included both PM_2.5_ and PM coarse. There were only minor changes in the effect size estimates for either pollutant, and both remained significant for all-cause mortality, although some of the cause-specific results had increased CIs that included no effect.

Table 4 shows the effects for PM_2.5_ and PM coarse by region. The number of cities varied in each region and by pollutant. For PM_2.5_ in the six regions, we have 47, 28, 17, 2, 3, and 15 cities, respectively. For PM coarse, we have 15, 11, 11, 2, 3, and 5 cities. The regions with dry climates and together with continental climate are the regions with the lower number of cities because they are not very populated, but they have the same number of cities for both PM_2.5_ and PM coarse. The effects of PM_2.5_ on all-cause mortality are similar for all regions except for the last (Mediterranean), which include California, Oregon, and Washington. The results were more varied for the specific causes of death, but the precision of the estimates was also less. There was a consistent trend for lower effects in the Mediterranean region for each cause as well.

In contrast, the pattern was different for coarse particles. First, there was considerably more variation in general in the all-cause mortality effects by region. Not only was the Mediterranean region different (as for PM_2.5_); there was no effect in the dry region as well. In addition, the effect size in the dry continental region was double that in the humid subtropical region for all-cause mortality and triple for CVD deaths.

The percentage of explained total variability differed between regions and between the two pollutants, with significant heterogeneity in the “warm summer, continental,” “hot summer, continental,” and “Mediterranean” regions when looking at all-cause mortality and PM_2.5;_ for PM coarse significant heterogeneity was found in “warm summer, continental” and “dry” regions.

## Discussion

In this national, multicity study we found a significant association between fine particulate air pollution and the risk of mortality for all causes, MI, CVD, stroke, and respiratory disease. We also found a significant association of coarse PM with daily deaths. Both effects were little changed after controlling for the other pollutant. There were several other features of our results worth noting.

These associations were higher during spring, which is consistent with the findings of the effects of PM_10_ on mortality by Zeka et al. (2006) but at variance with the findings of the National Morbidity, Mortality, and Air Pollution Study for PM_10_ (Peng et al. 2005), which found stronger effect during summer. It is also consistent with the report of Franklin et al. (2008), who showed that mean temperature in a given city had an inverted U-shaped association with the season-specific coefficient for PM_2.5_: Mild temperatures, which were associated with greater indoor penetration, were associated with higher PM_2.5_ effects, whereas both hot and cold temperatures were associated with lower effects. In our study, the higher effect sizes in the spring may reflect the same pattern.

In contrast to the PM_10_ association, where substantial regional differences have been reported, our analysis showed most climatic regions had very similar effect size estimates for PM_2.5_, except for the Mediterranean climatic region. However, the coarse particle effects varied much more, and the pattern of which regions were higher and lower differed between fine and coarse mode particles. This suggests that there are regional variations in the toxicity of coarse particles that require further study. It may be possible that coarse particles are coated with different substances in different regions, for example. In some previous studies, PM_10_ showed substantially lower effects and more regional heterogeneity than we see for PM_2.5_ and PM coarse. However, if the relative toxicity of PM fine and PM coarse varies differently by region, models fit with PM_10_ as the exposure index, which is essentially the sum of the two, may be effectively inducing measurement error in the exposure variable, which likely contributes to a downward bias in effect size, compared with treating them separately. Similarly, the different regional variability in effect size for fine and coarse mass may result in greater regional variability of PM_10_ coefficients.

One possible explanation for the lower effect in the Mediterranean region, which includes California, is more measurement error due to the extremely large counties in California, where people may live far away from the monitors. Moreover, in California there is substantial within-counties gradient in particle concentrations, as shown by Jerrett et al. (2005).

The magnitude of the PM_2.5_ risk estimates reported in our study are similar to the estimates from the two studies of Franklin and co-authors. In the first paper, Franklin et al. (2007) analyzed 27 cities that had PM_2.5_. The results from the case–crossover analysis for the previous day were a 1.21% (95% CI, 0.29 to 2.14%) increase in all-cause mortality, a 1.78% (95% CI, 0.20 to 3.36%) increase in respiratory-related mortality, and a 1.03% (95% CI, 0.02 to 2.04%) increase in stroke-related mortality with a 10-μg/m^3^ increase in previous day PM_2.5_. These results are generally comparable but slightly higher than ours, although we report results for a 2-day average.

In the second paper, Franklin et al. (2008) reported the association from the time series analysis by season for the 2-day averaged PM_2.5_ concentrations. They found a 0.74% (95% CI, 0.41 to 1.07%) increase in total mortality; a 0.47% (95% CI, 0.02 to 0.92%) increase in CVD; a 0.67% (95% CI, −0.21 to 1.07%) in stroke; and a 1.01% (95% CI −0.03 to 1.57%) increase in respiratory mortality. Again, these are generally comparable figures. In contrast, Dominici et al. (2007) examined the years 1999–2000 and reported a PM_2.5_ effect at lag 1 day of 0.29% (posterior interval, 0.01, 0.57) per 10-μg/m^3^ increase for all causes. This is lower than our estimate but was done using data for 2 years only. One other possible explanation for the difference is that Dominici et al. (2007) estimated an effect for PM_2.5_ in a single model for all seasons. In contrast, our analysis and the Franklin time-series analysis fit separate regressions in each city for each season. This allows for seasonal differences in the effect of day of the week terms and of the splines for temperature. Hence, it allows, for example, for different effects of a hot day in May versus August. In contrast, Dominici et al. used many more degrees of freedom to control for weather variables, but fit those curves for the entire year. Thus, control for confounding was different. In addition, we fit different effect size estimates for PM_2.5_ for each season. Although we averaged those estimates to obtain a yearly average effect, because that is relevant to a pollutant with an annual average standard, we began with different effects by season, which were significant. In contrast, the Dominici analysis effectively assumes the same size effect in each season. If that assumption is false, it could result in a type of measurement error such as attenuation in the effect size estimate, even for the annual average effect, because seasonal changes in toxicity of 1 μg/m^3^ of particles can be considered as a seasonal variation in measurement error [in the use of the same exposure metric, when the true exposure (toxicity weighted) is different]. Moreover, the differences in the effect sizes could be explained by differences in exposure metrics, where we also used the mean of lags 0 and 1 instead of lag 1 alone as exposure metric.

There is considerable toxicologic support for these findings. Animal experiments indicate that reactive oxygen species, which have established relevance in the pathogenesis of CVD and aging (Dhalla et al. 2000), are affected by particles, which represents one pathway for their cardiovascular and lung effects (Brook et al. 2004; Gurgueira et al. 2002; Nel 2005; Rhoden et al. 2004). Diesel particles increase oxidative stress in endothelial tissue, inducing the production of heme oxygenase-1, a rapid response part of the body’s defense system against oxidative stress (Furuyama et al. 2006). The viability of cell cultures of microvascular endothelial cells was impaired by diesel particles, with an accompanying large increase in induction of heme oxygenase-1 (Hirano et al. 2003).

The implication of oxidative stress mechanisms has epidemiologic support as well. A recent report showed that subjects who were *GSTM1* null or had the long variant of *HMOX-1* had enhanced effects of particles on heart rate variability, including a three-way interaction (Chahine et al. 2007). Rossner et al. (2008a, 2008b) examined bus drivers in Prague and reported increased levels of indicators of oxidative stress such as F-2 isoprostane and 8-hydroxydeoxyguanosine (8-OHdG) in drivers compared with controls. Particles, and particularly the metals on particles, have also been associated with an increased production of 8-OHdG (Chuang et al. 2007; Kim et al. 2004; Knaapen et al. 2002; Prahalad et al. 2000, 2001), including specifically exposure to traffic pollution (Chuang et al. 2003; Lai et al. 2005; Ma and Ma 2002; Tokiwa et al. 1999). 8-OHdG was also elevated in urban children compared with rural children (Tondel et al. 2005).

Other mechanisms have also been implicated. Particles have been shown to increase sICAM-1 (soluble intercellular adhesion molecule-1) and sVCAM-1 (soluble vascular adhesion molecule 1) in diabetics (O’Neill et al. 2007), a finding confirmed in a controlled human exposure chamber study (Salvi et al. 1999). Both animal and controlled human exposure studies have demonstrated that ambient particles can increase prothrombotic (clot-forming) activity and even induce thrombosis in acute exposures (Mills et al. 2007; Mutlu et al. 2007; Nemmar et al. 2003). In a relevant epidemiologic study, Baccarelli et al. (2007, 2008) reported an association of airborne particles with decreased clotting time as well as the risk of deep vein thrombosis. This is consistent with the results of a controlled exposure study to diesel particles, which reported increased ST depression (a sign of ischemic heart disease) and alterations in fibrinolytic capacity (the ability to break up clots that have formed in blood vessels) (Mills et al. 2007).

## Conclusions

In summary, there is a strong association of both fine and coarse particles with daily deaths. These associations are biologically plausible and, at the mean concentrations in the United States, suggest tens of thousands of early deaths per year, which could be avoided by reducing particle concentrations. Because coarse particles are not currently regulated by U.S. EPA and many power plants and pre-2007 diesel engines are grandfathered from having to retrofit controls to reduce fine particles, considerable public health improvement may be possible.

## Figures and Tables

**Table 1:** Combined results across 112 cities of the mortality PM_2.5_ association for the mean01 and for the mean01 by season.

	Percent (95% CI)	I^2^
All-cause mortality		
Overall	0.98 (0.75 to 1.22)	0.21*
Winter	0.56 (0.17 to 0.94)	0.14
Spring	2.57 (1.96 to 3.19)	0.32*
Summer	0.25 (−0.13 to 0.63)	0
Autumn	0.95 (0.56 to 1.34)	0.15*
CVD		
Overall	0.85 (0.46 to 1.24)	0.17*
Winter	0.70 (0.04 to 1.36)	0.11
Spring	2.18 (1.22 to 3.15)	0.22*
Summer	−0.03 (−0.75 to 0.69)	0.04
Autumn	0.92 (0.17 to 1.68)	0.22*
MI		
Overall	1.18 (0.48 to 1.89)	0.05
Winter	1.29 (−0.14 to 2.75)	0.16*
Spring	2.12 (0.53 to 3.74)	0.03
Summer	−0.03 (−1.46 to 1.42)	0
Autumn	1.24 (0.12 to 2.36)	0
Stroke		
Overall	1.78 (0.96 to 2.62)	0.10*
Winter	1.93 (0.34 to 3.54)	0.18*
Spring	2.04 (−0.02 to 4.13)	0.15
Summer	1.64 (0.05 to 3.26)	0
Autumn	1.69 (0.06 to 3.35)	0.19*
Respiratory		
Overall	1.68 (1.04 to 2.33)	0.10*
Winter	0.86 (−0.16 to 1.88)	0.06
Spring	4.62 (3.08 to 6.18)	0.10
Summer	0.78 (−0.49 to 2.06)	0
Autumn	1.45 (0.19 to 2.72)	0.14

Values are percent increase (95% CI) for 10-μg/m^3^ increase in PM_2.5_. I^2^ statistics and significance level (*p < 0.05) for heterogeneity. Mean01 is the mean of lags 0 and 1 (overall and by season).

**Table 2:** Combined results across 47 cities of the mortality PM coarse association for the mean01 and for the mean01 by season.

	Percent (95%CI)	I^2^
All-cause mortality		
Overall	0.46 (0.21 to 0.71)	0.26*
Sum distributed lag	0.31 (0.00 to 0.63)	0
Winter	−0.14 (−0.48 to 0.20)	
Spring	1.01 (0.47 to 1.57)	0.41*
Summer	0.57 (−0.03 to 1.18)	0.29*
Autumn	0.44 (−0.02 to 0.89)	0.25*
CVD		
Overall	0.32 (0.00 to 0.64)	0.05
Winter	−0.47 (−1.04 to 0.10)	0
Spring	0.95 (0.01 to 1.90)	0.41*
Summer	1.00 (0.34 to 1.67)	0
Autumn	0.16 (−0.36 to 0.68)	0
MI		
Overall	−0.12 (−0.80 to 0.56)	0.05
Winter	−1.23 (−2.38 to −0.07)	0
Spring	0.60 (−1.06 to 2.28)	0.22
Summer	0.87 (−0.51 to 2.27)	0
Autumn	−0.23 (−1.40 to 0.95)	0.02
Stroke		
Overall	0.84 (0.07 to 1.62)	0.06
Winter	0.38 (−1.32 to 2.12)	0.13
Spring	0.74 (−0.71 to 2.21)	0.07
Summer	−0.02 (−1.87 to 1.87)	0.07
Autumn	1.54 (0.34 to 2.76)	0
Respiratory		
Overall	1.16 (0.43 to 1.89)	0.19*
Winter	0.64 (−0.86 to 2.17)	0.27*
Spring	2.56 (0.99 to 4.16)	0.33*
Summer	−0.05 (−1.34 to 1.25)	0.004
Autumn	1.18 (0.10 to 2.26)	0.04

Values are percent increase (95% CI) for 10-μg/m^3^ increase in PM_2.5_. I^2^ statistics and significance level (*p < 0.05) for heterogeneity. Mean01 is the mean of lags 0 and 1 (overall and by season).

**Table 3:** Percent increase (95% CI) in mortality for 10-μg/m^3^ increase in PM coarse and PM_2.5_ for the mean01 across the 47 cities; two-pollutant model and single-pollutant model results for PM_2.5_ in 47 cities.

	PM_2.5_	PM coarse
All-cause mortality	0.77 (0.43 to 1.12)	0.47 (0.21 to 0.73)

	0.94 (0.65 to 1.22)	

CVD	0.61 (0.05 to 1.17)	0.29 (−0.04 to 0.61)

	0.97 (0.51 to 1.43)	

MI	0.75 (−0.12 to 1.63)	0.04 (−0.72 to 0.81)

	1.18 (0.43 to 1.93)	

Stroke	0.82 (−0.21 to 1.86)	0.71 (0.02 to 1.41)

	1.96 (0.88 to 3.07)	

Respiratory	1.63 (0.69 to 2.59)	1.14 (0.43 to 1.85)

	1.92 (1.08 to 2.78)	

Two-pollutant models are shaded. Mean01 is the mean of lags 0 and 1.

**Table 4:** Percent increase (95% CI) in mortality for 10-μg/m^3^ increase in the mean01 PM_2.5_ and PM coarse, combined by regions.

	PM_2.5_		PM coarse	
	Percent (95% CI)	I^2^	Percent (95% CI)	I^2^
All-cause mortality				

Humid, subtropical, and maritime	1.02 (0.65 to 1.38)	0.06	0.53 (0.18 to 0.88)	0.03
Warm summer, continental	1.19 (0.73 to 1.64)	0.18*	0.77 (0.14 to 1.40)	0.37*
Hot summer, continental	1.14 (0.55 to 1.73)	0.27*	0.87 (0.47 to 1.26)	0
Dry	1.18 (−0.70 to 3.10)	0	−0.02 (−1.11 to 1.10)	0.57*
Dry, continental	1.26 (−0.21 to 2.76)	0.11	1.11 (0.11 to 2.11)	0
Mediterranean	0.50 (0.00 to 1.01)	0.40*	−0.46 (−0.95 to 0.02)	0.24

CVD				

Humid, subtropical, and maritime	0.78 (0.05 to 1.51)	0.17*	0.21 (−0.34 to 0.77)	0
Warm summer, continental	1.43 (0.67 to 2.19)	0.15	0.88 (−0.13 to 1.90)	0.34*
Hot summer, continental	0.43 (−0.53 to 1.40)	0.17	0.71 (0.03 to 1.39)	0
Dry	3.11 (−0.02 to 6.33)	0	0.28 (−1.29 to 1.88)	0.33
Dry, continental	1.67 (−0.75 to 4.16)	0	0.70 (−1.26 to 2.69)	0
Mediterranean	0.16 (−0.46 to 0.79)	0.12	−0.15 (−0.87 to 0.58)	0.16

MI				

Humid, subtropical, and maritime	0.97 (−0.29 to 2.26)	0	0.82 (−0.88 to 2.56)	0.22*
Warm summer, continental	1.50 (0.05 to 2.97)	0.07	−0.45 (−1.73 to 0.84)	0
Hot summer, continental	0.64 (−0.96 to 2.28)	0	0.40 (−1.05 to 1.87)	0
Dry	4.25 (−2.38 to 11.33)	0	−2.16 (−4.82 to 0.57)	0
Dry, continental	0.60 (−7.42 to 9.32)	0.17	5.09 (0.31 to 10.09)	0.12
Mediterranean	1.85 (−0.66 to 4.41)	0.34*	−1.18 (−2.28 to −0.08)	0

Stroke				

Humid, subtropical, and maritime	2.94 (1.59 to 4.32)	0	1.62 (0.37 to 2.89)	0
Warm summer, continental	1.85 (0.04 to 3.69)	0.13	1.03 (−1.41 to 3.53)	0.26*
Hot summer, continental	0.77 (−1.77 to 3.38)	0.23*	0.86 (−1.23 to 3.01)	0.22
Dry	1.82 (−6.98 to 11.45)	0.17	0.34 (−2.43 to 3.18)	0.01
Dry, continental	2.49 (−2.32 to 7.53)	0	0.32 (−3.75 to 4.56)	0
Mediterranean	0.95 (−0.66 to 2.59)	0.23*	0.07 (−1.14 to 1.31)	0

Respiratory				

Humid, subtropical, and maritime	0.91 (−0.25 to 2.08)	0.03	2.16 (1.14 to 3.20)	0
Warm summer, continental	2.12 (0.89 to 3.36)	0.04	1.67 (0.03 to 3.33)	0.16
Hot summer, continental	3.36 (1.95 to 4.79)	0	2.09 (0.80 to 3.40)	0
Dry	5.81 (−0.04 to 12.00)	0.22	−2.55 (−5.39 to 0.37)	0.49*
Dry, continental	−0.31 (−5.89 to 5.61)	0.26	1.81 (−1.93 to 5.70)	0.32
Mediterranean	1.06 (−0.36 to 2.50)	0.38*	−0.68 (−1.78 to 0.43)	0.08

I^2^ statistics and significance level (*p < 0.05) for heterogeneity. Mean01 is the mean of lags 0 and 1.
